# Data Augmentation and Synthetic Data Generation in Rare Disease Research: A Scoping Review

**DOI:** 10.3390/medsci13040260

**Published:** 2025-11-06

**Authors:** Rebecca Finetti, Bianca Roncaglia, Anna Visibelli, Ottavia Spiga, Annalisa Santucci

**Affiliations:** 1 Department of Biotechnology, Chemistry and Pharmacy, University of Siena, 53100 Siena, Italy; rebecca.finetti@student.unisi.it (R.F.); bianca.roncaglia@unisi.it (B.R.); ottavia.spiga@unisi.it (O.S.); 2 Centro della Scienza e della Tecnica, Polo Universitario Grossetano, Via Ginori 41, 58100 Grosseto, Italy; 3 Industry 4.0 Competence Center ARTES 4.0, Viale Rinaldo Piaggio, 56025 Pontedera, Italy

**Keywords:** data augmentation, rare diseases, synthetic data, machine learning

## Abstract

Background: Rare diseases represent a significant research challenge due to the limited availability of data, small patient cohorts, and heterogeneous phenotypes. Data augmentation and synthetic data generation are increasingly adopted to mitigate these limitations. Methods: This scoping review maps the application of data augmentation and synthetic data generation methods as strategies to address these limitations. A total of 118 studies published between 2018 and 2025 were identified through PubMed, Scopus, and Electronics Engineers (IEEE) Xplore. Results: Imaging data headed the field, followed by clinical and omics datasets. Classical augmentation, mainly geometric and photometric transformations, emerged as the most frequent approach, while deep generative models have rapidly expanded since 2021. Rule- and model-based methods were less common but demonstrated high interpretability in small datasets. Conclusions: Overall, these techniques enabled dataset expansion and improved model robustness. However, both approaches require rigorous validation to confirm biological plausibility. Together, these methods can transform data scarcity from a barrier into a driver of methodological innovation, enabling more inclusive rare disease research.

## 1. Introduction

Rare diseases are conditions that affect a relatively small number of individuals compared to the general population [1], and their rarity presents specific challenges and issues, affecting over 350 million patients worldwide with approximately 7000 distinct conditions. Access to highly specialized, quality care faces two main obstacles: late diagnosis and limited therapeutic development [2]. The diagnostic pathway for patients with rare diseases is extremely challenging. On average, it takes six years from the onset of symptoms to receive an accurate pathological diagnosis due to several factors, including the low prevalence of these diseases, insufficient numbers of specialists with the necessary scientific expertise, limited access to specialist care, and inadequate research infrastructure [3]. In addition, the development of targeted therapies is hindered by data scarcity and small patient cohorts, which limit the research into pathophysiological mechanisms and therapeutic options. Meanwhile, rare diseases are now increasingly recognized as a critical public health issue, leading to international networks to raise awareness and drive research progress in the field [4]. At the same time, artificial intelligence (AI) has been progressively adopted to address these challenges [5,6] though its success relies on access to large datasets, which is challenging in the context of rare diseases. Small sample sizes, heterogeneous phenotypes, and fragmented data collections increase the risk of model overfitting and poor generalizability [7]. Therefore, insufficient data is one of the most significant limitations of data-driven approaches in rare disease research [8,9]. Promising strategies to address this limitation are data augmentation and synthetic data generation, which involve methods that artificially expand or enrich datasets either through the modification of existing samples or through the creation of new synthetic ones. Originally prevalent in computer vision, these techniques have more recently been applied to temporal [10], biomedical [11,12], and multi-omic data [13]. More recently, nonlinear and hybrid generative architectures have been proposed, including the integration of deep learning techniques with tensor decomposition frameworks for advanced neuroimaging analysis [14]. Such approaches enable greater robustness, generalisation, and resilience in downstream predictive models.

In the field of rare diseases, where recruiting patients and acquiring data is resource-intensive, augmentation can help to overcome small sample size issues, together with class imbalance, and restricted variability in clinical and biological data [15]. Furthermore, the generation of synthetic data may facilitate the simulation of disease progression, accelerate biomarker discovery, and support the design of clinical trials [16,17,18]. Despite their potential, augmentation and synthetic data generation are rarely used in rare disease research, with approaches often borrowed from other fields and hardly validated under the unique constraints of rare and ultra-rare conditions. Questions remain regarding the biological plausibility of augmented and synthetic data, how it can be integrated into clinical pipelines, and how its impact on model performance can be evaluated. The purpose of this review is to map the state of the art on data augmentation and synthesis within the context of rare diseases. Our objectives are to (1) describe the range of augmentation and synthetic data generation techniques adopted to date, (2) clarify their purposes and areas of application, and (3) discuss existing limitations and unresolved methodological issues. Bringing together the available evidence, we intend to provide both biomedical researchers and data scientists with guidance on how these approaches can help overcome one of the major obstacles in rare disease research: the limited availability of robust and representative datasets.

## 2. Materials and Methods

This review was performed in accordance with the PRISMA (Preferred Reporting Items for Systematic Reviews and Meta-Analyses) guidelines [19]. In particular, the following five-stage methodological framework was employed [20]: (1) Identify research questions, (2) Identify relevant studies, (3) Study selection, (4) Data Charting, (5) Collate and report results. This meta-analysis has been registered in PROSPERO (registration number CRD420251179956). The study protocol can be found on the same registry.

### 2.1. Literature Search and Selection

We comprehensively searched three databases, PubMed, Electronics Engineers (IEEE) Xplore, and Scopus, for candidate articles. Eligible records had to meet the following criteria: (1) focused on data augmentation or synthetic data in the context of rare diseases, (2) published or made publicly available between 1 January 2010, and 31 August 2025, (3) written in English. The general search strategy combined terms related to “data augmentation” or “synthetic data” with “rare disease”. For PubMed, the major repository of life science and biomedical publications, we also paired 9784 individual rare disease names from the Orphanet database (Table S1) with the terms “data augmentation” and “synthetic data”. To evaluate the robustness of this approach, the results were compared against a set of known relevant articles identified during preliminary screening. The initial search was conducted in August 2025, and the search strings for each database are reported in Table 1. In total, 2864 candidate articles were retrieved.

Once all the candidate articles were obtained, we screened titles, abstracts, and full texts. Citation management and initial screening were conducted using Rayann [21]. Exclusion criteria were: (1) not published in a peer-reviewed journal or conference proceeding, (2) not original research (e.g., editorials, reviews), (3) not involving human subjects, (4) not addressing data augmentation, synthetic data, and rare diseases. These exclusion criteria were applied to ensure the inclusion of relevant and original studies. Non-peer-reviewed and non-original research works were excluded to guarantee scientific reliability, to avoid duplication of evidence, and to focus on primary data. Studies not involving human subjects were excluded to maintain consistency with the clinical and translational scope of the review. Papers not addressing data augmentation, synthetic data, or rare diseases were excluded as they fell outside the main research question. Each publication’s title and abstract were independently screened by a minimum of two researchers. Any discrepancies or ambiguities were resolved through collegial discussion and consensus among all researchers.

### 2.2. Full-Text Analysis and Metadata Extraction

After the initial screening of abstracts, 150 publications were identified as relevant to the aim of this scoping review and were selected for further analysis based on their full texts. However, 148 publications were selected, as one article was retracted from the public record and the other was not available online, with no response received from the authors. A minimum of two researchers independently screened each of the 148 publications. During this process, the main data elements included (1) publication year, (2) study focus, (3) rare diseases, (4) data type, (5) data size (before), (6) data size (after), (7) method. Table 2 summarizes the metadata elements.

## 3. Results

**Figure 1:**
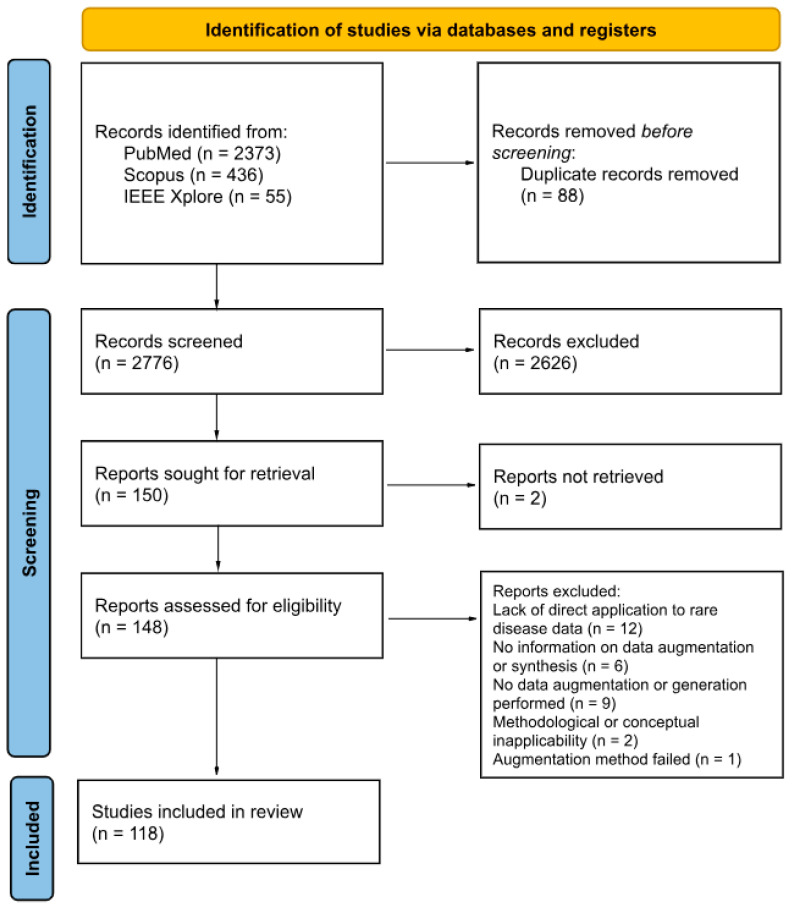
PRISMA 2020 flow diagram for systematic reviews based on the publications on this topic.

The article selection process was performed in accordance with the PRISMA Preferred Reporting Items for Systematic Reviews and Meta-Analyses (PRISMA) flow diagram guidelines, as illustrated in Figure 1. Of the 2776 articles initially identified after deduplication, 148 were subject to full-text screening. The rigorous application of the inclusion criteria, coupled with a detailed examination of each publication, resulted in 118 publications being included in this scoping review. Articles were excluded for the following reasons: (1) Lack of direct application to rare disease data; (2) Absence of data augmentation or generation; (3) Insufficient methodological detail regarding the use of augmentation or synthetic data generation; (4) Methodological or conceptual inapplicability (framework-oriented papers not relevant to real-world rare disease data augmentation); and (5) Failed augmentation methods (reported unsuccessful or unreliable attempts). The titles and authors of the included publications are provided in Table S2, which summarizes the main characteristics of each study, including data type, study focus, rare disease investigated, and the augmentation or generation methods applied.

### 3.1. Temporal Trends of Publication

**Figure 2:**
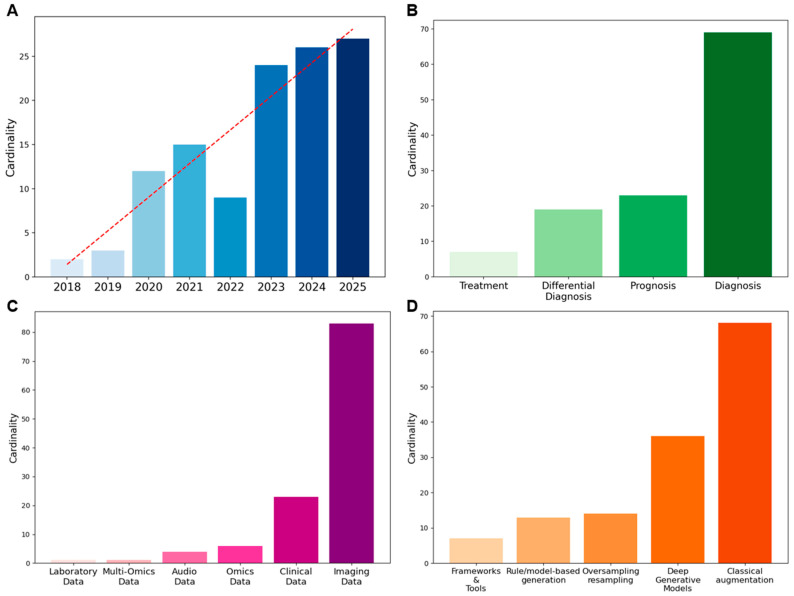
Overview of metadata: (**A**) Temporal trend of publications, (**B**) Distribution of study focus, (**C**) Data types employed, (**D**) Methods applied for data augmentation/synthetic data generation.

Figure 2A shows the yearly evolution of the included publications. Although our search covered the period from 2010 onwards, no relevant articles to this scoping review were retrieved before 2018. The absence of augmentation and synthetic data techniques in the biomedical domain is not surprising, given that these techniques have only recently been adopted from computer vision and other data-rich fields. The first 2 publications appeared in 2018 (1.7%), followed by a gradual increase: 3 in 2019 (2.5%), 12 in 2020 (10.2%), and 15 in 2021 (12.7%). After a short decline in 2022 with only 9 articles published (7.6%), interest grew exponentially, reaching 24 in 2023 (20.3%), 26 in 2024 (22.0%), and 27 in 2025 (22.9%).

### 3.2. Study Focus

Among the 118 articles, several analytical tasks are represented (see Figure 2B). Of these, 69 (58.5%) focus on diagnosis, 23 (19.5%) on prognosis, 19 (16.1%) on differential diagnosis, and 7 (5.9%) on treatment. Overall, diagnosis is the most frequent focus of studies in augmentation and synthetic data generation for rare diseases.

### 3.3. Rare Diseases Classification

Data augmentation and synthesis techniques have been applied to more than eighty rare diseases identified in the reviewed literature. The most represented domain includes hematological malignancies, particularly acute lymphoblastic leukemia, acute myeloid leukemia, and acute promyelocytic leukemia, which collectively account for the largest share of studies. Hepatic and oncological disorders, such as hepatocellular carcinoma and cholangiocarcinoma, also appear frequently, reflecting the widespread use of imaging data in these contexts. Genetic and metabolic diseases represent another substantial group, comprehending conditions like cystic fibrosis, Duchenne muscular dystrophy, and sickle cell disease, which are often explored in multi-disease or cross-cohort frameworks. Neurological and neuro-oncological conditions, including glioblastoma, meningioma, and craniosynostosis, are discussed in several contributions, together with systemic or autoimmune disorders such as systemic lupus erythematosus, systemic sclerosis, and Kawasaki disease. A smaller but noteworthy portion of studies addresses ophthalmological and endocrine diseases, including retinopathy of prematurity and thyroid carcinomas. However, the process of clear-cut classification remains challenging, as many rare conditions span multiple organ systems or etiological groups.

### 3.4. Data Type and Data Size

As illustrated in Figure 2C, the majority of the studies used imaging data, which covered 70.3% of the total, followed by clinical data (19.5%). Omics data accounted for 5.1%, while audio data represented 3.4%. Less frequent categories included multi-omics (0.8%) and laboratory data (0.8%). Quantitative information on dataset size before and after augmentation, when available, is detailed in Table S2. However, only 57% of the reviewed papers reported explicit numerical values for either the initial or augmented dataset, and less than one-third provided both. Where reported, imaging datasets increased on average about threefold, while non-imaging data showed broader variability. The remaining studies did not specify the number of samples generated, confirming a general lack of standardized reporting in this area.

### 3.5. Method

In this review, methods were categorized according to their data transformation principle. This conceptual distinction was adopted to ensure terminological consistency across studies, where definitions sometimes overlapped or were inconsistently applied. Classical augmentation methods, primarily geometric and photometric transformations, emerged as the most frequently applied strategies (49.3%), followed by deep generative models (DGMs) (26.1%). Rule/model-based synthetic data generation approaches accounted for 9.4%, while oversampling and resampling methods represented 10.1%. Frameworks and tools were less common, comprising 5.1% of the applications. The distribution of methods is displayed in Figure 2D. To provide an overview, Table 3 summarizes the main methodological differences between data augmentation and synthetic data generation, highlighting their typical scales of dataset expansion, main techniques, strengths, and limitations.

## 4. Discussion

This scoping review highlights the rapid increase in the use of data augmentation and synthesis in rare disease research over the past five years. Distinct patterns and challenges emerge when the distribution of methods is mapped across different data types, disease categories, and research focuses.

### 4.1. Data Augmentation

**Figure 3:**
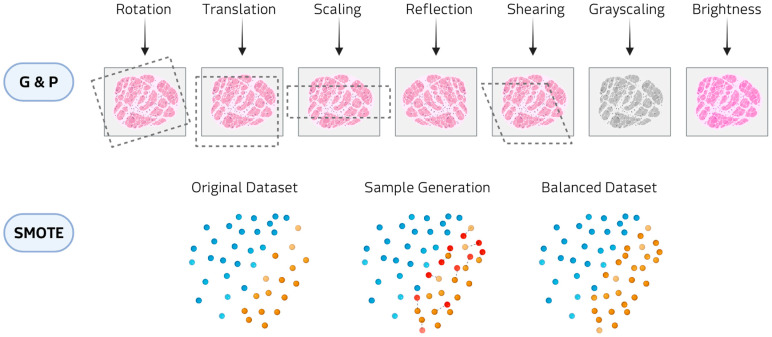
Examples of data augmentation methods: Geometric and photometric transformations (G&P) at the top and SMOTE at the bottom.

The main data augmentation approaches include classical augmentations and oversampling/resampling methods (Figure 3).

In classical augmentation, geometric and photometric transformations constitute the most widely employed class in biomedical imaging, applied in almost 48% of the reviewed studies. Geometric transformations (e.g., rotations, translations, reflection, and shearing) modify the spatial configuration of the image, thereby simulating variability in patient positioning, tissue orientation, or magnification during acquisition. Photometric transformations (e.g., adjustments of brightness and contrast, and color perturbations) alter the pixel intensity distribution to mimic differences in staining protocols, illumination conditions, or scanner settings. These approaches have been reported to be applied in hematological diseases, where digital histopathology slides are expanded to reduce class imbalance [22,23,24,25,26,27,28,29,30,31,32]. Within classical augmentation, patch-based resampling methods are augmentation techniques that split medical images into smaller units, either as overlapping 3D cubes or slice-wise stacks. These approaches have been applied in rare tumor imaging [33,34] and rare neurological diseases [35]. This strategy allows models to focus on localized structural and textural features, reducing memory constraints associated with full-volume data.

Oversampling techniques, such as Synthetic Minority Over-sampling Technique (SMOTE) and Adaptive Synthetic Sampling (ADASYN), are widely applied to clinical imaging and omics data. SMOTE generates artificial samples by selecting a minority-class instance and interpolating new points along the line segments connecting it to its nearest minority-class neighbors. ADASYN builds on this principle by adaptively concentrating the generation of synthetic samples in regions of the feature space where class imbalance is more pronounced or classification is more challenging. In hematological malignancies, these methods have been employed to counteract imbalanced patient cohorts and improve predictive modeling [36]. In oncological contexts, SMOTE has been used to generate balanced datasets for imaging studies [37,38,39]. Beyond cancer, oversampling strategies have also been applied to metabolic and inherited disorders, where the scarcity of cases and controls poses major challenges [40,41]. In audio and signal processing domains, oversampling augmentations have been employed to enrich datasets for speech-related and movement disorders [42,43,44]. These transformations alter the temporal or frequency characteristics of the signals in ways that mimic natural variability in voice production, sensor noise, or recording conditions. Although technically lightweight, they are effective at enlarging small cohorts.

### 4.2. Synthetic Data Generation

**Figure 4:**
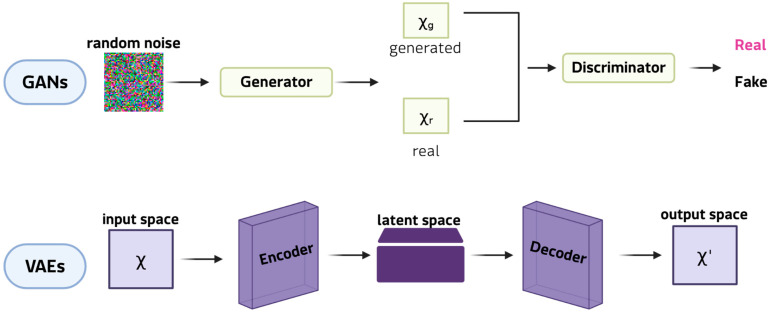
Examples of DGMs: GANs at the top and VAEs at the bottom.

Although less common than classical augmentation, the generation of synthetic data has received considerable attention since 2021, largely due to advances in DGMs. DGMs (Figure 4) allow realistic yet innovative samples to be generated that retain essential patterns and correlations, while introducing sufficient variability to enrich training sets [45].

This ability is particularly valuable in the study of rare diseases, where a lack of patient data often restricts the reliability of subsequent analyses. Among DGMs, generative adversarial networks (GANs) are the most innovative and widely adopted framework for synthetic data generation. In their classic formulation, a GAN comprises two neural networks: the generator, which learns to produce synthetic samples from random noise vectors, and the discriminator, which determines whether a given sample is genuine or artificial. Through this antagonistic training cycle, the generator progressively captures the statistical properties of the original dataset, enabling the creation of new data points that closely resemble real observations [46]. This mechanism makes GANs particularly well-suited to expanding limited datasets, as they can model complex, high-dimensional distributions and reproduce subtle patterns that simpler augmentation strategies often lose. Key extensions include conditional GANs, which generate class-specific images; CycleGANs, which are effective in cross-modal translation (e.g., Magnetic resonance imaging (MRI) and computed tomography (CT) scans, and histopathological staining); and StyleGANs, which separate global and local features to produce realistic, detailed results [47]. These models can synthesise entirely new images that capture complex pathological heterogeneity, thereby expanding datasets beyond the scope of simple transformations. For instance, GAN-generated retinal scans of rare ophthalmic diseases [48] have doubled the size of training sets and enhanced the performance of classification in diagnostic pipelines. Applications also include the generation of MRI scans for small glioblastoma cohorts [49]. Variational autoencoders (VAEs), on the other hand, are used to generate synthetic patient records for clinical and tabular data. VAEs are particularly promising in omics research because their probabilistic latent space provides a structured and continuous representation of high-dimensional data. VAEs learn to reconstruct input data by compressing it into a lower-dimensional latent space and then sampling from this distribution. This framework enables smooth interpolation between samples, making VAEs ideal for modelling disease progression or transitions between phenotypic states. For instance, VAEs have been employed to simulate transcriptomic profiles of Niemann-Pick disease [40], generating virtual cohorts for comparing biomarker discovery workflows and testing analytical robustness. Rule- and model-based approaches, which generate synthetic data by explicitly modeling probabilistic dependencies between variables, represent a smaller class of generation methods. These include Bayesian networks and, in particular, dynamic Bayesian models that capture temporal relationships among clinical variables to simulate virtual patient trajectories. Recent applications to amyotrophic lateral sclerosis (ALS) have demonstrated that dynamic Bayesian networks can generate realistic longitudinal data, preserving conditional dependencies over time and enhancing downstream predictive modeling when combined with transfer learning [50,51]. Compared with deep generative models, these approaches offer higher interpretability and reliability on small datasets, although they reproduce a limited range of variability. This strategic use of data generation is particularly pertinent for bioinformatics applications in rare tumors, a field where molecular insights are often limited by data sparsity and feature instability. While current rare cancer bioinformatics studies [52,53] have successfully developed robust models by focusing on refined feature selection and deep learning architectures to mitigate these intrinsic data issues, generative strategies offer a complementary solution.

### 4.3. Advantages and Limitations

Data augmentation and synthetic data generation both address the challenge of small and imbalanced datasets in rare disease research; however, they differ in their technical scope, strengths, and limitations. Data augmentation is generally simpler, computationally inexpensive, and easier to validate. It works well when the data structure lends itself to transformations that do not compromise its meaning, for instance, rotating or scaling an MRI slice does not change the underlying pathology. This explains why data augmentation is prevalent in diseases where the use of images for diagnosis and prognosis is essential, such as hematological diseases and tumors. With simple transformations, it is therefore possible to expand datasets and balance small clinical cohorts without requiring specialist infrastructure or expertise. In terms of clinical feasibility, classical augmentation has high interpretability; the resulting data’s source is clear, which significantly increases clinician trust and simplifies integration into existing diagnostic workflows. This accessibility explains why classical augmentation continues to dominate the field. However, data augmentation can only modify what is already available, and if the original data are biased or incomplete, the resulting dataset will simply reproduce those shortcomings on a larger scale. It is also important to note that augmentation is unable to generate truly novel, unobserved patient phenotypes, thus limiting its capacity to comprehensively capture the heterogeneity that characterises numerous rare diseases.

Synthetic data generation, on the other hand, opens possibilities that augmentation alone cannot offer. Beyond technical utility, it also offers a practical advantage because it can produce artificial yet realistic patient records for clinical trials, potentially accelerating discovery when recruiting real patients is difficult. At the same time, synthetic data carries important limitations, since it is more powerful but technically demanding. Technical applicability is a major challenge for DGMs, which often suffer from instability, require significant computational power (GPU clusters) for training on complex datasets, and demand sophisticated hyperparameter tuning [54]. They also risk generating biologically implausible outputs [55]. Despite the consistent improvement of downstream models that incorporate DGM-generated data, few studies provided explicit technical validation of the generated samples themselves. In most studies, augmented or synthetic data were evaluated only by improvements in model performance, while independent clinical assessment or external benchmarking is limited. To improve clinical feasibility, validation needs to move beyond performance metrics to include measures of clinical utility, such as fidelity (how closely synthetic data matches the real data distribution) and diversity (the extent of novel, yet plausible, data generated). An additional limitation emerging from this review concerns the inconsistent quantitative reporting of dataset size before and after augmentation. Despite the inclusion of this information in our metadata extraction, more than 40% of the publications did not provide sufficient detail to compare expansion rates properly, limiting the comparability and reproducibility of reported results. Moreover, ethical and regulatory issues remain, especially regarding whether and how synthetic data can be accepted in diagnostic workflows or clinical research pipelines. A key step toward the adoption of these methods is the development of reliable validation frameworks that can ensure the clinical value of augmented and synthetic datasets. Validation should not be restricted to numerical performance metrics but should also include contextual evaluation to assess the coherence of generated data with disease mechanisms and clinical manifestations.

Cross-source comparisons and collaborative benchmarking are essential for identifying biases and verifying reproducibility. This approach aligns with the latest regulatory science for transparent synthetic health data usage. For example, the FDA’s internal programs addressing synthetic data and generative AI in medical device evaluation [56], EMA’s roadmap for ethical data sharing [18], and IRDiRC’s emphasis on data standardization and sharing in rare disease projects [57].

## 5. Conclusions

This review provides the first overview of how data augmentation and synthetic data generation are being applied across different rare disease domains. The proposed framework can help biomedical and computational scientists to select suitable techniques, to prioritize validation efforts, and to define benchmarks for reproducible and interpretable models in rare disease studies. Methodological innovation, combined with careful evaluation, can transform data scarcity into a catalyst for more robust and inclusive research. The increasing use of augmentation and generative strategies highlights the commitment to overcoming the limitations of small and imbalanced datasets. While classical augmentation provides accessibility and stability, synthetic approaches offer unprecedented opportunities to simulate biological variability. In the future, the impact of these methods will depend on rigorous validation, transparent reporting, and alignment with ethical and regulatory frameworks. Closer collaboration among data scientists, clinicians, and regulators, together with open and interoperable infrastructures, will be essential to ensure that models and datasets align with biological and clinical priorities. Furthermore, the widespread inconsistencies identified in this review highlight the urgent need for a common reporting framework for augmentation and synthesis studies. To enhance reproducibility and comparability, such a framework must, at a minimum, require transparent reporting of the following: (1) the size and composition of the initial dataset, including class distribution; (2) the size of the final dataset after augmentation/synthesis, with the expansion rate specified per class; (3) the precise details of the algorithm(s) used; and (4) the metrics used to validate the quality, diversity and biological plausibility of the generated data beyond model performance alone.

## Figures and Tables

**Table 1:** Database-specific search strings.

Database	Search Strings
PubMed	data augmentation AND rare disease data augmentation AND one of 9784 individual rare disease names synthetic data AND rare disease synthetic data AND one of 9784 individual rare disease names
IEEE	data augmentation AND rare disease synthetic data AND rare disease
Scopus	data augmentation AND rare disease synthetic data AND rare disease

**Table 2:** Metadata extracted from each article.

Metadata	Definition	Categories or Range
Publication year	Article publication year	Between 2018 and 2025
Study focus	Focus of the article on data augmentation and/or synthetic data generation for rare diseases	Diagnosis Treatment Prognosis Differential diagnosis
Rare disease	Disease(s) or group of diseases investigated in the article	
Data type	Where the inputs for the model(s) are derived from	Imaging data Audio data Clinical data Laboratory data Omics data Multi-omics data
Data size (before)	Quantity related to a particular type of data sample before augmentation and synthesis	
Data size (after)	Quantity related to a particular type of data sample after augmentation and synthesis	
Method	The methodology used for data augmentation and/or the generation of synthetic data	Classical augmentation Deep generative models Oversampling techniques Rule/model-based generation approaches Frameworks and tools

**Table 3:** Comparison between data augmentation and synthetic data generation methods.

	Data Augmentation	Synthetic Data Generation
Scale of expansion	2–4× increase over the original dataset	Up to 10× or more, depending on model type and training data
Main techniques	Geometric and photometric transformations, patch resampling, and oversampling	Deep generative models
Strengths	Simple, interpretable, computationally efficient, low risk of artifacts	Can model complex variability, enhance diversity
Limitations	Limited to existing data variability; may amplify original biases	Computationally demanding; potential for artifacts; validation and interpretability remain challenging
Common applications	Image-based diagnosis and prognosis, small clinical cohorts	Simulation of disease variability, virtual cohorts, and data sharing in restricted domains

## Data Availability

The original contributions presented in this study are included in the article/Supplementary Material. Further inquiries can be directed to the corresponding authors.

## Supplementary Materials

The following supporting information can be downloaded at: https://www.mdpi.com/article/10.3390/medsci13040260/s1, Table S1: Supplementary data 1; Table S2: Supplementary data 2.

## Abbreviations

The following abbreviations are used in this manuscript:

GANs	Generative adversarial networks
VAEs	Variational autoencoders
AI	Artificial intelligence
IEEE	Electronics Engineers
SMOTE	Synthetic Minority Over-sampling Technique
ADASYN	Adaptive Synthetic Sampling
G&P	Geometric and photometric transformations
DGMs	Deep generative models
MRI	Magnetic resonance imaging
CT	Computed tomography
